# Estimation of health-related utility (EQ-5D index) in subjects with seasonal allergic rhinoconjunctivitis to evaluate health gain associated with sublingual grass allergen immunotherapy

**DOI:** 10.1186/1477-7525-12-99

**Published:** 2014-06-13

**Authors:** Chris D Poole, Christian A Bannister, Jakob Nørgaard Andreasen, Jens Strodl Andersen, Craig J Currie

**Affiliations:** 1 Cochrane Institute of Primary Care and Public Health, Cardiff University, Cardiff, Wales, UK; 2 ALK, Bøge Allé, Copenhagen, Hørsholm, Denmark

## Abstract

**Background:**

Grass allergen immunotherapy (AIT) reduces symptom severity in seasonal allergic rhinoconjunctivitis (ARC) but its impact on general health-related utility has not been characterised for the purposes of economic evaluation. The aim of this study was to model the preferred measure of utility, EQ-5D index, from symptom severity and estimate incremental quality adjusted life years (QALYs) associated with SQ-standardised grass immunotherapy tablet (GRAZAX®, 75,000 SQ-T/2,800 BAU, ALK, Denmark).

**Methods:**

Data were analysed from five consecutive pollen seasons in a randomised placebo controlled trial of GRAZAX®. Binomial and Gaussian mixed effects modelling related weekly EQ-5D index score to daily symptom and medication scores (DSS & DMS respectively). In turn, daily EQ-5D index was estimated from ARC symptoms and medication use.

**Results:**

DSS and DMS were the principal predictors of ‘perfect’ health (EQ-5D = 1.000; binomial) and ‘imperfect’ health (EQ-5D < 1.000; Gaussian). Each unit increase in DSS and DMS reduced the odds of ‘perfect’ health (EQ-5D = 1.000) by 27% and 16% respectively, and reduced ‘imperfect’ health by 0.17 and 0.13, respectively. Gender remained the only other significant main fixed effect (Male odds ratio [OR] = 1.82). Incremental estimated EQ-5D index utility for GRAZAX® was observed from day -30 to day +70 of the pooled pollen season; mean daily utility for GRAZAX® = 0.938 units (95%CI 0.932-0.943) vs. 0.914 (0.907-0.921) for placebo, an incremental difference of 0.0238 (p < 0.001). This translates into an incremental 0.0324 Quality Adjusted Life Years over the five year study period.

**Conclusions:**

ARC symptoms and medication use are the main predictors of EQ-5D index. The incremental QALYs observed for GRAZAX® may not fully describe the health benefits of this treatment, suggesting that economic modelling may be conservative.

## Background

The increasing prevalence of atopic disease is a significant global health concern with up to 20% of Western adults suffering from allergic rhinoconjunctivitis (ARC) [1-3]. Although direct health care costs may be relatively modest compared to some other chronic disease (on average £49 per person per annum in the UK [4] after adjustment for inflation [5]), indirect costs such as work and school absenteeism and reduced workplace productivity are recognised to be considerable. For instance, it has been estimated that 3.5 million working days are lost annually in the United States due to this condition [6], and in Sweden the total annual cost of lost productivity is €_2008_2.7 billion [7].

Specific allergen immunotherapy potentially offers notable benefits over conventional symptomatic management, most notably clinical tolerance to allergen exposure sustained beyond the initial period of treatment [8]. Following extensive demonstration of both efficacy and tolerability [9-15], the SQ-standardized, containing *Phleum pratense* (timothy grass) pollen extract (GRAZAX® 75,000 SQ-T/2,800 BAU, ALK, Denmark)) is approved in Europe for disease modifying treatment of grass pollen–induced rhinoconjunctivitis in adults and children.

Economic evaluation of health technologies is an integral part of the post-approval reimbursement process. Most favoured methods of comparison involve evaluation of value for money in the form of cost-utility analyses (CUA) which measures health in terms of quality-adjusted life years (QALYs). QALYs quantify patients’ health-related utility on a scale from 0 (dead) to 1 (perfect health) allowing the health consequence of any clinical condition to be compared [16]. The most widely accepted method for determining QALYs is the EuroQoL-5D (EQ-5D) instrument [17]. The EQ-5D describes the patients’ health state using five dimensions: 1. Mobility, 2. Self-care, 3. Usual activities, 4. Pain/Discomfort and 5. Anxiety/Depression. Each of these dimensions has a number of potential responses. These are then transformed into a single index by applying a formula that attaches weights to each of the response levels in each dimension [18]. The weighting formulae are based on the valuation of EQ-5D health states from general population samples, and reflect the health preferences of their country of origin [19].

Rhinoconjunctivitis symptoms can vary rapidly in affected individuals in tandem with daily variation in pollen counts during the spring and summer months. When completing the EQ-5D, respondents are instructed to “indicate which statements best describe [their] own health state today” [17]. However clinical ARC studies (including those for GRAZAX) never record daily EQ-5D observations.

The purpose of this study was explore whether a model of daily health-related utility using EQ-5D derived data from randomised trials would more accurately reflect quality of life variation than weekly observations, and to use these data to determine the long-term utility benefit of GRAZAX® versus placebo in combination with the symptom relieving drugs taken normally by sufferers.

## Methods

### Study subjects

Details of the randomized, parallel group, double-blind, placebo-controlled phase-3 trial conducted according to the Declaration of Helsinki at 43 centers in 7 countries, assessing the efficacy and safety of grass allergen immunotherapy tablet 75,000 SQ-T (GRAZAX®; 75,000 SQ-T/2,800 BAU, ALK, Denmark) in subjects with seasonal grass pollen-induced rhinoconjunctivitis have been published previously (ClinicalTrials.gov number: NCT00227279) [11,13,20].

The study was conducted from 2005 to 2009, across 51 sites in 8 European countries (Austria, Denmark, Germany, Italy, The Netherlands, Spain, Sweden, and the United Kingdom). A total of 634 participants were randomised to either receive once-daily GRAZAX® or a placebo tablet similar in taste, smell and appearance. Double-blind treatment started at least 16 weeks before the planned start of the grass pollen season and continued throughout the pollen season. The start of each grass pollen season was defined as the first day of three consecutive days with grass pollen count of 10 grains/m^3^ or more with the end of the season defined as the last day in the last occurrence of three consecutive days with pollen count of more than 10 grains/m^3^. Patients were treated for three years and were followed for a further two years post treatment completion.

Main inclusion criteria were as follows: age 18 to 65 years; a clinical history of moderate-to-severe grass pollen-induced allergic rhinoconjunctivitis of two years or more requiring treatment during the grass pollen season; positive skin prick test result (wheal diameter ≥3 mm) and serum specific IgE (IgE CAP class ≥2) to *Phleum p*. Main exclusion criteria were as follows: forced expiration volume (FEV_1_) less than 70% of the predicted value; a clinical history of symptomatic seasonal allergic rhinitis/asthma due to tree or weed pollen potentially overlapping the grass pollen season; a clinical history of significant active perennial allergic rhinitis/asthma caused by an allergen to which the participant was regularly exposed; previous immunotherapy within the last five years; a history of anaphylaxis or angioedema; and pregnancy.

### Rhinoconjunctivitis symptom and medication scores

Each day for the full five years of the trial using an electronic diary, six rhinoconjunctivitis symptoms were measured on a scale from 0 to 3, with the following values: no symptoms (score = 0), mild symptoms (score = 1), moderate symptoms (score = 2), or severe symptoms (score = 3). The symptoms scored were: runny nose, blocked nose, sneezing, itchy nose, gritty feeling/red/itchy eyes, and watery eyes. The total daily rhinoconjunctivitis symptom score (DSS) was calculated for each participant as the sum of all individual symptom scores. The maximal total daily rhinoconjunctivitis symptom score was 18 arbitrary units. DSS was treated as a pseudo-continuous variable.

Symptom relieving medications used for the treatment of rhinoconjunctivitis were administered to all participants during the grass pollen seasons of the trial. The medications were to be used as required and entered in the diary by the subject. Each medication was scored according to symptom control (from 1 to 6 arbitrary units) as follows: desloratadine, 5 mg, up to 1 tablet daily (up to 6 points per day); olopatadine eye drops, 1.0 mg/mL, up to 1 drop per eye twice daily (up to 6 points per day); budesonide nasal spray, 32 mg per puff, up to 2 puffs per nostril twice daily (up to 8 points per day); and prednisone, 5 mg per tablet, up to 10 tablets per day (up to 16 points per day). The maximum daily medication score (DMS) was 36; however, if the recommended dose was exceeded, the actual score was used [20]. DMS was also treated as a pseudo-continuous variable.

### EQ-5D index

With regard to this specific analysis and not the pivotal trial, the primary endpoint was the EQ-5D index, calculated from weekly EQ-5D survey responses. In this multinational study there was a requirement to express utility by a single value set as (mixing value sets from each respective study would have made interpretation impossible) therefore we followed the advice of the EuroQoL group that “In the absence of a suitable candidate, adopt the most robust valuation set (the UK TTO set).” [18,19].

### Model derivation and development

Subjects in the study cohort were primarily younger adults ― average age was 34 years ― with low co-morbidity, aside from a history of seasonal allergic conjunctivitis. Therefore it was expected that a high proportion of the EQ-5D responses would indicate a ‘perfect’ health state (‘11111’ or 1.000 unit), creating a highly skewed distribution. For this reason EQ-5D index values were analysed using a ‘two-part’, mixed modelling approach. In the first stage, the response variable (EQ-5D index) was modelled as a binary variable indicating ‘perfect health’ (1.000) or ‘imperfect health’ (<1.000). Responses indicating ‘imperfect health’ were then evaluated in a second stage of modelling where the EQ-5D index was used as a pseudo-continuous variable. Two-part modelling has been shown to be less biased than other limited dependent variable regression models for dealing with the bounded nature of health utility data including censored Tobit and censored least absolute deviation (CLAD) regression methods [21-23]. Selection of predictors, both fixed and random effects, for inclusion in the models was informed using backward stepwise selection methods where covariates below the specified significance level (p < 0.05/t < 2) were identified and removed.

### Binomial modelling of ‘perfect’ health

In the first stage of the modelling process, the relationship between the EQ-5D index (as a binary response variable) with observed predictor variables was determined using a mixed effects logistic model. Gender, age, treatment arm, diagnosis of asthma, daily rhinoconjunctivitis symptom score, daily medication score, a combined symptom/medication score, and the number of days with severe symptoms were considered as fixed effects. Year of treatment and study country were considered as random effects to adjust for variation due to differences between years and countries.

To identify subjects for entry into the next modelling stage, a threshold was calculated for use with the probability of ‘perfect health’ predicted by the logistic model. Using the predicted probabilities from the logistic model, a classification threshold (or cut-off) was calculated. This was obtained by considering all possible thresholds (0.001 – 0.999) and selecting the threshold that yielded the maximum combined specificity and sensitivity. In the absence of domain information to indicate that being in perfect health and identified as being in perfect health, is of different importance to not being in perfect and identified as not being in perfect health we gave, specificity and sensitivity equal weighting in calculation of the threshold. All observations with a predicted probability of ‘perfect health’ above the threshold were assigned a predicted EQ-5D index score of 1.000; those below the threshold were identified for inclusion in the second modelling stage.

### Continuous modelling of those with imperfect health

In the second stage of the modelling process, the relationship of the EQ-5D index (as a pseudo-continuous response variable) with observed predictor variables was determined using a generalized linear, mixed model. The dependent variable, pseudo-continuous EQ-5D index was transformed using a cubic function to achieve normally distributed residuals. The same candidate predictors as in the first (binomial) stage model were also considered for fixed and random effects.

Empirically we reasoned that an individual’s EQ-5D response to the fixed-effects acting upon them would be consistent and therefore the error terms within a subject were correlated. We further assumed an autoregressive process would operate under the premise that past values have an effect on current values. We considered that a first order process (AR (1)), meaning that the current value is based on the immediately preceding value, would adequately represent this implied relationship. Residuals were assumed to be normally distributed and the explanatory power of predictors as fixed or random effects were compared using Akaike’s Information Criterion (AIC) where appropriate. All analyses were carried out in R (version 2.14.0) [24].

### Comparison of utility

The two-part model and probability threshold developed using the weekly diary data were then retrospectively applied to the daily diary data in order to calculate the individual QALYs for the study period. Due to the annual variations in pollen season intensity observed during the trial [20], a final pooled five-year estimate of incremental QALYs between the treatment arms was made by calculating mean simulated EQ-5D index for each pollen day relative to the designated start of each respective pollen season, defined as the first day of three consecutive days with grass pollen count of 10 grains/m^3^ or more [12]. To account for any observable treatment effect outside the study-defined pollen season, a pooled estimate of incremental QALYs was made from the first day where three consecutive between-group *t*-tests had p ≤ 0.05 to the last day where three consecutive tests had p > 0.05.

## Results

Six hundred and thirty-four participants were included in the trial at randomisation (316 active and 318 placebo). Five hundred forty-six participants completed the first season of the trial. When the trial was extended to further four years, 351 participants continued in the extension (189 active, 162 placebo). These participants were a representative subset of the population originally included in the trial [12]. Two hundred and thirty-eight participants (135 active, 103 placebo) completed the planned five-year trial duration. There were 17,599 weekly observations of EQ-5D, DSS and DMS used in model development and 137,792 daily observations used in the EQ-5D simulations. The baseline characteristics were similar between the treatment groups and they have been reported previously [13].

### Binomial modelling

After variable selection, the final model utilised gender, symptom score and medication score as fixed effects, and subject and year of treatment as random effects to predict ‘perfect’ health. Predictors that were considered but deemed as contributing too little to the model and thus omitted from the final model included age, treatment arm, diagnosis of asthma, combined symptom/medication score, the number of days with severe symptoms and study country. A summary of the first-stage, mixed effects logistic model developed using weekly diary data is provided in Table 1. Symptom score, followed by medication score were the most ‘significant’ predictors of ‘perfect’ health, reflecting a decrease in odds of ‘perfect’ health of 27% and 16% for each unit increase in score for DSS and DMS, respectively (odds ratios of 0.7286 and 0.8406). There was also a small but significant interaction effect (odds ratio of 1.0119) between DSS and DMS. Gender was a ‘significant’ predictor, being male increased the odds of ‘perfect’ health by 82% (odds ratio of 1.8169). Comparing the predicted probabilities with observed proportions yielded a NMSE of 0.39673, suggesting acceptable model performance.

**Table 1:** **Summary of mixed effects logistic model** (**stage 1**)

**Fixed effects**	**Estimate**	**Std. error**	**z value**	**Pr (>|z|)**	**Odds ratio**
(Intercept)	3.84611	0.24695	15.57	< 2e-16	-
Sex (male vs. female)	0.59713	0.24315	2.46	0.01406	1.8169
Symptom score	-0.31662	0.02505	-12.64	< 2e-16	0.7286
Medication score	-0.17369	0.03039	-5.72	1.1e-08	0.8406
Interaction (symptoms and medication)	0.01185	0.00309	3.84	0.00013	1.0119

By jointly optimising sensitivity and specificity, a threshold probability of 0.960 was calculated. Using the threshold and predicted probabilities from the first stage (binomial) model; weekly observations were classified as either ‘perfect health’ or ‘imperfect health’.

### Continuous modelling

Weekly observations classified as ‘imperfect health’ were used in the development of the second-stage generalized linear mixed model summarised in Table 2. After variable selection, the fixed effects of symptom and medication score and a subject random effect were sufficient to predict the EQ-5D index in subjects identified as ‘imperfect’ health in the previous (binomial) modelling stage. Predictors that were considered, but deemed as contributing too little to the model and thus omitted from the final model, included gender, age, treatment arm, diagnosis of asthma, combined symptom/medication score, the number of days with severe symptoms, year of treatment and study country. A DSS and DMS interaction term was tested but did not contribute ‘significantly’ to the model and was therefore omitted. A unit increase in symptom and medication score were associated with a decrease of predicted EQ-5D summary score of 0.17 and 0.13, respectively.

**Table 2:** **Summary of generalized linear mixed model** (**stage 2**)

**Fixed effects**	**Estimate**	**Std. error**	***t *** **value**	**Estimate (original scale)**
(Intercept)	0.516469	0.007012	73.7	0.8023
Symptoms score	-0.004478	0.001053	-4.3	-0.1648
Medication score	-0.001986	0.000891	-2.2	-0.1257

### Daily EQ-5D index simulation

**Figure 1:**
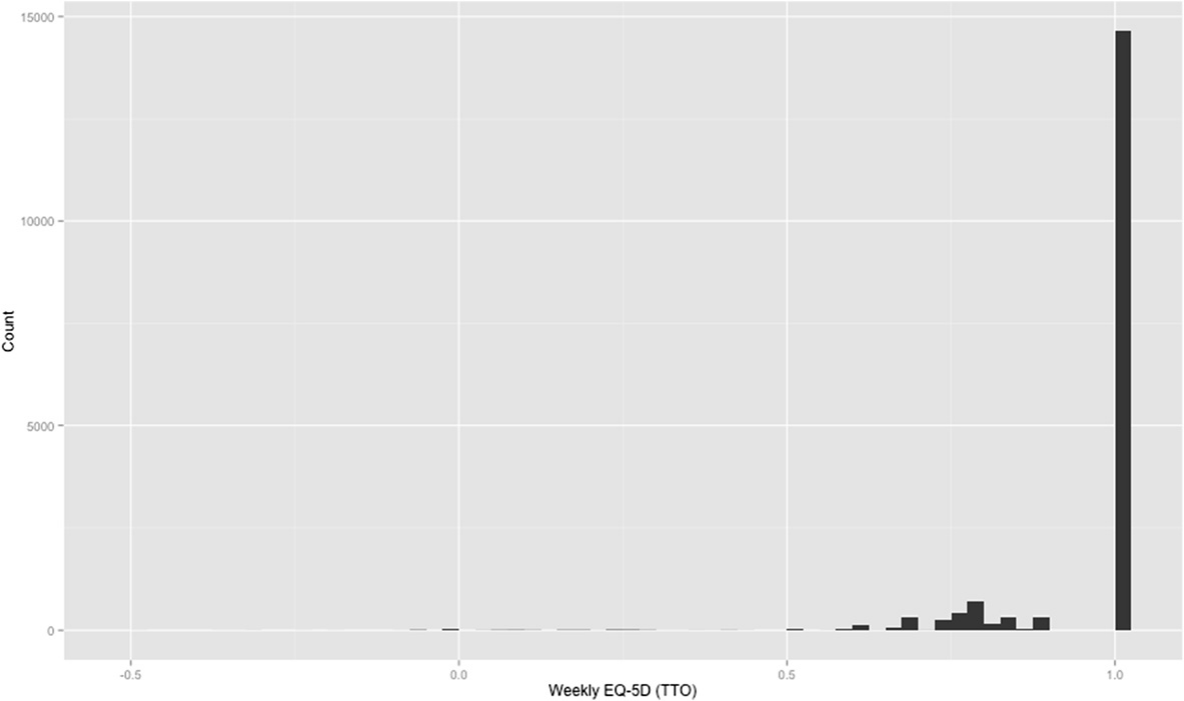
**Histogram for weekly observed EQ**-**5D utility scores (TTO).**

Table 3 reports mean absolute error (MAE), mean squared error (MSE) and root mean squared error (RMSE) of predicted compared to actual utility scores by EQ-5D utility range for the two-part model using the weekly diary data. Table 3 also reports mean observed and mean predicted values to give an indication of over- or under-prediction, whilst presenting means in this way can be misleading, the pros outweigh the cons in this instance. MAE and MSE reported in Table 3 suggest that the model predicts well overall and for the milder health states, but over predicts the value for more severe health states. This poorer performance in the more severe states may be associated with the distributions of actual EQ-5D utility values in Figure 1, where there are relatively very few weekly observations in the more severe health states. There are currently no guidelines as to when estimation errors are and are not acceptable, but a systematic review of mapping studies [25] has reported MAEs from 0.0011 to 0.19, and RMSEs from 0.084 to 0.2. The MAE of the two-part model (Table 3) was outside of this range for the relatively severe health states (EQ-5D < 6.0) where there were very few observations (Figure 1), however overall MAE the model was 0.073.

**Table 3:** **Mean absolute error**, **mean squared error and root mean squared error of predicted compared to actual utility scores by EQ**-**5D utility range using the weekly diary data**

**EQ**-**5D utility score**	**Mean absolute error**	**Mean squared error**	**Root mean squared error**	**Observed mean**	**Predicted mean**	**N (%)**
<0.5	0.723	0.570	0.755	0.117	0.841	161 (0.92%)
0.5-0.599	0.275	0.086	0.293	0.561	0.836	78 (0.44%)
0.6-0.699	0.151	0.031	0.176	0.670	0.820	497 (2.82%)
0.7-0.799	0.085	0.017	0.130	0.749	0.834	684 (3.89%)
0.8-0.899	0.088	0.012	0.111	0.830	0.855	1542 (8.76%)
0.9-1.0	0.061	0.013	0.114	1.000	0.939	14637 (83.17%)
Full Index	0.074	0.019	0.138	0.956	0.923	17599 (100%)

The two-part model developed from the weekly diary data was then applied to the daily diary data. Using the first-stage model and associated threshold, 87,725 (63.7%) of the 137,792 observations in the daily data were classified as ‘perfect health’ and assigned an EQ-5D summary score of 1.000. The second-stage model was then applied to the 50,067 (36.3%) daily observations that were classified as ‘imperfect health’ from the daily data, assigning them a pseudo continuous EQ-5D index score. Where the probability of ‘perfect health’ was less than the cut-off, the continuous model was used to simulate the degree of ‘imperfect health’ for that data point.

### Incremental QALYs

**Figure 2:**
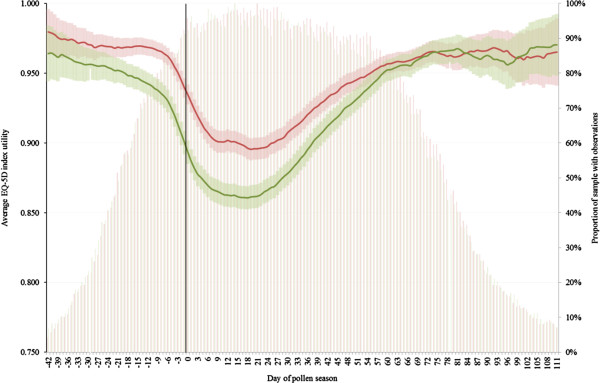
**Primary axis**: **predicted pooled mean daily utility for study period**, **stratified by treatment arm (GRAZAX®, red line; placebo, green line [with 95% CI]).** Secondary axis: proportion of study sample with observations by day of pollen season (GRAZAX®, red bars; placebo, green bars).

**Figure 3:**
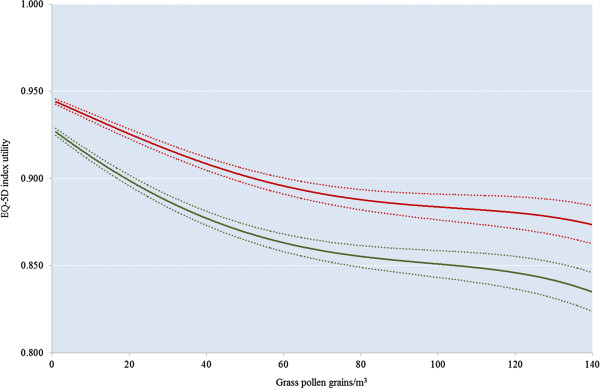
**Predicted daily utility by pollen count**, **stratified by treatment arm (GRAZAX®, red: placebo, green; dotted lines represent 95% CIs).**

There was temporal and geographical variability in the start dates and durations of grass pollen seasons between sites and between years. The earliest and latest starts of grass pollen seasons were 4^th^ April and 14^th^June, respectively. The earliest and latest ends of grass pollen season were 7^th^ June and 27^th^ August, respectively. The duration of the grass pollen seasons ranged from 15 to 116 days across all sites and all years. For this reason, the mean utility value is presented by relative day of a particular grass pollen season rather than by calendar date. The mean EQ-5D index (with 95% CI) for the five-year, pooled day of the pollen season, stratified by treatment arm is given in Figure 2. The onset of incremental utility benefit of GRAZAX® over placebo occurred at day minus 30 of the pollen season and continued until day +70. Between these dates the mean daily utility for GRAZAX® patients was 0.938 units (95% CI 0.932 to 0.943) compared with an average of 0.914 (95% CI 0.907 to 0.921) for placebo, an incremental difference of 0.0238 units (p < 0.001).Figure 3 shows improvement in the mean predicted EQ-5D for GRAZAX® and placebo subjects across the spectrum of pollen count days. The area between the curves during the 100-day benefit period defined above equates to a mean annual increment of 0.0065 Quality Adjusted Life Years (QALYs), or 0.0324 QALYs over the five year study period.

## Discussion

It was possible to determine an overall difference in health-related utility between those treated with GRAZAX® and those treated with placebo.

This study is the most comprehensive formal investigation of the relationship between health-related utility and allergic rhinoconjunctivitis symptom severity. Use of a two-part modelling approach reduced the adverse impact of the highly skewed multimodal distribution of EQ-5D index, especially in a population of relatively young adults with low co-morbidity, among whom the majority of EQ-5D health states indicated ‘perfect’ health. The adjusted limited dependent variable mixture model (ALDVMM) approach recently proposed by Hernández et al. [26] provides a credible alternative to the two-part modelling approach used in the current study. Unfortunately Hernández et al. did not compare their approach with ‘two part’ modelling, therefore we opted for the latter which has been shown to perform consistently better than censored regression. It is important to note that the current study is not a comparative assessment of statistical methodology, and the adoption of ALDVMM would have been non-trivial to due to some of issues with estimation of mixture models, such as local optima. Until there are libraries and/or functionality in the mainstream statistical packages these methods will be out of reach for many researchers. Despite a modest sample size, the use of repeated measures mixed modelling ensured efficient use of the repeated observations. Our results showed unequivocally that the strongest predictors of the EQ-5D index ― health-related utility ― in this population were rhinoconjunctivitis symptom score followed by medication score.

Although variable length of pollen season from year-to-year could confound the observed QoL benefits, the presentation of a five-year ‘average’ pollen season would mitigate this, and for the purposes of health economic evaluation an ‘average’ pollen season is a reasonable construct. This would be confounded if some systematic change in the length of pollen seasons had been evident during the study, however this was not the case.

The five-year, pooled incremental simulated utility was strikingly similar to the reported increment of 0.0287 units taken from the first year’s data in the same trial [27], supporting the assumption of maintained QALY gain for GRAZAX® used in cost-effectiveness modelling with a longer time horizon than the initial treatment phase. Our findings confirm that the beneficial impact of GRAZAX® on health-related utility seen in the first year of treatment was carried forward through all five years observed here. Interestingly, the pattern of pooled data also seemed to indicate a pre-season utility benefit for patients receiving GRAZAX® compared to patients receiving placebo. This is reasonable given that the definition of a pollen season for the purposes of the study would not preclude ‘out-of-season’ individual days with a high pollen count. However, the correlation of EQ-5D to the rhinoconjunctivitis symptom and medication scores was determined for the scales used in the GRAZAX trials, meaning that a separate correlation has to be evaluated for trials using other symptom and medication scorings. The original hypothesis for this study was that weekly measurement of EQ-5D may not fully capture the disutility associated with a disease which has a highly variable symptom course that varies daily according to meteorological variation and therefore might underestimate the benefit of primary prevention treatment. Having conducted the experiment using justifiable modelling methods, we were unable to refute the null hypothesis.

Some commentators might observe that the incremental utility seen in this study was lower than the lowest estimate of the minimum clinically important difference (MCID) for the EQ-5D index (0.033) [28]. This would in fact create a misleading picture of the quality of life benefits of GRAZAX® experienced by patients as the relative insensitivity of EQ-5D to changes in rhinoconjunctivitis symptom severity is documented. In a comparison of patients with seasonal allergic conjunctivitis and matched controls, Pitt et al. [4] found that despite significant differences (p < 0.001) in all domains of the Rhinoconjunctivitis Quality of Life Questionnaire, only the pain/discomfort domain of the EQ-5D differed modestly in these patients. A repeat of the same study design in Spain reported similar effect sizes [29].

The relative insensitivity of the EQ-5D to rhinoconjunctivitis symptom severity is perhaps to be expected. The EQ-5D does not record impairment in important QoL domains adverse affected by rhinoconjunctivitis symptoms such as sleep quality and workplace productivity. The Spanish study (mainly moderate-severe symptoms) found that over half of the subjects experienced poor sleep quality, while one-fifth suffered excessive diurnal somnolence [30]. A systematic review of studies comparing rhinoconjunctivitis symptoms with HRQoL impairment found that sleep, daily activities, physical and mental status and social functioning were all impacted consistently [31]. Within the current study population the relative difference in sleep problems between GRAZAX® and placebo (Δ35%; p = 0.0009) was the highest of any domain of the Rhinoconjunctivitis Quality of Life questionnaire (RQLQ) [14].

In addition, it has been demonstrated that conditions (including AR) with lower symptom severity exhibit ceiling effects which limits the discriminant ability of EQ-5D [32]. Stull et al recommend inclusion of another utility measure that can capture the lower-end of symptom severity spectrum as a useful strategy for establishing health utility in these patients. The Short Form 36-item (SF-36) generic QoL questionnaire has been shown to have acceptable discriminative properties in rhinoconjunctivitis [33], suggesting that its associated utility index the SF-6D might be a better choice of utility instrument for this disease group. This finding is supported by a longitudinal survey of young adults with seasonal rhinoconjunctivitis in which significant differences in the following SF-36 domains were observed between baseline outside the pollen season and peak season observations: physical functioning, physical role, bodily pain and vitality [34]. Vitality has been recognised to be a unique contributor to SF-6D in certain clinical situations [35]. In their review of the statistical properties of five different utility instruments (including the EQ-5D), Palta et al. found the SF-6D to be least prone to ceiling effects, and showed least variation in responsiveness across the range of values [36].

## Conclusion

Rhinoconjunctivitis symptoms and associated medication use are the main predictors of EQ-5D index utility in an adult population with seasonal grass pollen-induced rhinoconjunctivitis. We have demonstrated that use of grass allergen immunotherapy tablet 75,000 SQ-T exhibits long-term QoL benefits over placebo.

## Competing interests

All authors declare: financial support for the submitted work by ALK; JNA and JSA are employed by ALK; CB has received a PhD scholarship from the MRC and the EPSRC; CDP has been employed by Pharmatelligence, a research consultancy receiving funding from pharmaceutical companies (including ALK); CJC has received research grants from various health-related organizations including Abbott, Astellas, Diabetes UK, the Engineering and Physical Sciences Research Council, the EASFD, Ferring, GSK, Lilly, the Medical Research Council, Medtronic, MSD, the National Health Service, Pfizer, Sanofi-Aventis, Shire, and Wyeth and consults for Amylin, Aryx, Astellas, Boehringer Ingelheim, BMS, Diabetes UK, Eisel, Ferring, GSK, Ipsen, Lilly, Medtronic, MSD, Pfizer, Sanofi-Aventis, Takeda, and Wyeth; no other relationships or activities that could appear to have influenced the submitted work.

## Authors’ contributions

The authors contributed the following: CDP, JNA, JSA and CJC conceived the study. CDP, JNA and JSA contributed to study design. CB and CDP analyzed the data. CB and CDP drafted the paper. CB, CDP, JNA, JSA and CJC interpreted the results. CB, CDP, JNA, JSA and CJC were involved in revising the paper. CJC had overall responsibility for the study and is overall guarantor. All authors read and approved the final manuscript.
